# What patients with cancer require from their clinicians to enable uptake of exercise as part of cancer care: A mixed methods study

**DOI:** 10.1007/s11764-025-01779-4

**Published:** 2025-03-25

**Authors:** Prue Cormie, Ashleigh Bradford, Lisa Guccione, Peter Martin, Meg Chiswell, Christopher M. Doran, Mei Krishnasamy

**Affiliations:** 1 Department of Health Services Research, Peter MacCallum Cancer Centre, 305 Grattan Street, Melbourne, VIC 3000 Australia; 2 Sir Peter MacCallum Department of Oncology, The University of Melbourne, VIC, Australia; 3 School of Medicine, Faculty of Health, Deakin University, VIC, Australia; 4 Centre for Research in Patient Communication, Faculty of Health, University of Southern Denmark, Copenhagen, Denmark; 5 Healthcare Communication Collective, VIC, Australia; 6 Centre Cluster for Resilience and Wellbeing, Appleton and Manna Institutes, Central Queensland University, Brisbane, QLD Australia; 7 Department of Nursing, The University of Melbourne, VIC, Australia; 8 Victorian Comprehensive Cancer Centre Alliance, VIC, Australia

**Keywords:** Exercise, Physical activity, Cancer, Oncology, Healthcare communication, Advice

## Abstract

**Purpose:**

Despite robust evidence and a series of evidence-based guidelines stating the benefits of exercise for cancer patients, uptake remains low. This study aimed to explore and describe what patients with cancer require from their clinicians to enable uptake of exercise as part of cancer care.

**Methods:**

Concurrent mixed methods design. Participants included adult cancer patients. Online questionnaires and semi-structured telephone interviews explored patient preferences for receiving information about the role of exercise in cancer care. Quantitative data were analysed using standard descriptive statistics and an interpretive descriptive approach was used to inform qualitative analysis.

**Results:**

Participants included 456 cancer patients. A randomly selected sub-set of 30 patients completed an interview. Many participants (*n* = 280/61.6%) reported discussion with clinicians as the way they mostly preferred to receive exercise information. Receiving exercise information shortly after being diagnosed and before starting treatment (*n* = 186/41.1% and *n* = 90/19.9%) were the timepoints mostly preferred for being informed about exercise. Information that was personalised and described exercise as being important to their cancer care was reported to be more likely to influence exercise behaviour. Clinician actions/provision of resources ranked as most helpful were: referral to cancer-specific exercise specialist (*n* = 330/76.6%) or program (*n* = 310/71.8%), and written exercise recommendations from a doctor/nurse (*n* = 234/54.3%).

**Conclusions and Implications for Cancer Survivors:**

Cancer patients would be more likely to consider exercise as part of their cancer care if their clinicians initiated an informed conversation about exercise, introduced early in the care continuum, using personalised and cancer-specific messaging, that was supported by referral to cancer-specific exercise services.

**Supplementary Information:**

The online version contains supplementary material available at https://doi.org/10.1007/s11764-025-01779-4.

## Introduction

Many clinical trials across various cancer types, stages and treatments have established exercise as an efficacious adjunct therapy that counteracts common treatment-related side-effects experienced by cancer patients [1–5]. Exercise (i.e. regular, moderate-intensity aerobic and resistance exercise) has been shown to significantly reduce the severity of cancer-related fatigue, anxiety and depression; improve physical function and quality of life; while having a low risk of adverse events [1–5]. This body of evidence has led major cancer organisations such as the American Society of Clinical Oncology [6] and Clinical Oncology Society of Australia [7] to call for exercise to be incorporated into routine cancer care [6–12]. Specifically, these organisations recommend cancer clinicians advise patients to exercise in line with evidence-based guidelines to mitigate treatment-related side effects [6–12].

Cancer clinicians are uniquely placed to facilitate patient exercise behaviour as trusted experts whose advice has the potential to modify exercise attitudes and beliefs [13–18]. However, clinicians face a multitude of challenges to incorporate exercise recommendations into routine practice including structural, knowledge, skill and patient factors [19–21]. Additionally, clinicians have little evidence available to help optimise the effectiveness of their exercise advice [13–15]. While screening and referral algorithms have been developed [22–28], it remains unclear how and when exercise information should be given, what information matters to patients and what actions/resources patients want to receive from their cancer care team. Patient adherence is highly correlated with person-centred communication [29–31] but there is a paucity of research investigating how patients want their clinicians to discuss exercise, or that supports clinicians to align their information provision with patients’ needs [22–28].

Complex factors influence exercise behaviour among cancer patients with considerable research examining exercise participation barriers, facilitators, preferences and behaviour change techniques [32–38]. Comparatively little research has focused on the provision of exercise information by clinicians [39–41], despite widespread calls in the literature for clinicians to recommend exercise [6–8, 22, 23]. Patients with cancer consistently report interest in receiving information about exercise [33–35] but few studies have reported on patient preferences for the delivery of exercise recommendations [42]. Consequently, it is unclear what patients need from cancer clinicians to consider exercise as part of their plan of care. To address this gap in evidence, this study explored patient preferences for receiving information about the role of exercise in cancer care.

## Methods

A concurrent mixed methods design was used involving online questionnaires and semi-structured telephone interviews. Mixed-methods reporting guidelines (Mixed Methods Reporting in Rehabilitation and Health Sciences [MMR-RHS]) were adhered to in the development and reporting of the study [43]. The action, actor, context, target, time (AACTT) framework for specifying behaviour of healthcare providers informed the focus of the questions developed for, and the analytical approach applied to this study [44]. The project was approved by Peter MacCallum Cancer Centre Human Research Ethics Committee (HREC/74874) and all participants provided informed consent.

### Participants

Patients with cancer currently receiving or within three years of completing treatment with curative or palliative intent were invited to participate. Although a convenience sampling approach was used, strategies to encourage recruitment from a diverse sample of participants included recruitment through a public cancer hospital serving metropolitan and regional patients and engaging community organisations serving diverse groups of patients with cancer. Participants were recruited using promotional materials that were displayed in the waiting rooms of the cancer hospital; provided by a member of multidisciplinary cancer care teams; and distributed by community-based cancer organisations. A quick-response code provided access to the online participant information sheet. Informed consent was provided online before participants completed the anonymous questionnaire. Participation was voluntary with no incentives offered or compensation provided. Patients who had a palliative care inpatient admission within the last three months, were unable to read/speak English without an interpreter, or were less than 18 years old were excluded. All participants were asked to indicate willingness to take part in one audio-recorded telephone interview at the end of the questionnaire. A randomly selected sub-sample of those who indicated willingness to take part in an interview were recruited by a research assistant by direct email using strata to obtain 1:1 balance for treatment status (yes/no currently having disease modifying treatment), sex (female/male), age (< 65 years ≥), as well as exercise levels in proportion to the rates observed in the survey (1:3 do/don’t meet exercise guidelines). The participant information sheet and consent form were provided electronically by a research assistant and written informed consent obtained prior to the interview.

### Quantitative data collection and analysis

In the absence of validated instruments, study-specific questionnaire items were developed to explore patient preferences for receiving information about the role of exercise in cancer care. This approach is similar to previous research examining exercise programming preferences among patients with cancer [32–36]. The questionnaire development process involved review of the literature (e.g. studies included in [32–36]), stakeholder consultation and iterative discussions among authors (described in Online Resource 1). Questionnaire items underwent pilot testing with eight patients with cancer with feedback gathered from interviews guiding enhancements (Online Resource 1). Items were also reviewed by eight cancer nurses to obtain a broad perspective of patient needs expressed in routine practice with feedback collected through think aloud methodology guiding refinements [45] (Online Resource 1). Brief, evidence-based exercise information was provided in the questionnaire preamble to inform participants of the evidence underpinning exercise guidelines (Online Resource 2). This information was provided to allow participants to consider the type, format and timing of exercise information that would best suit their individual needs irrespective of prior knowledge about exercise in cancer care (i.e. standardised base of knowledge). The questionnaire items explored patient preferences for receiving exercise information as a component of cancer care. The AACTT framework [44] was used to identify preferences across domains: ‘who’ (Actor domain) should ‘provide exercise information’ (Action domain); in ‘what format’ (e.g. discussion, information booklet; Context domain) and ‘when’ should exercise information be provided (Time domain) to the patient (Target domain) were explored. Response options included seven-point Likert scales (strongly agree to strongly disagree; very much to not at all) as well as ranking (best to worst). The sequence response items were presented in ranking questions was randomised to mitigate any potential positional bias.

Standard clinical and demographic information was obtained through self-report using established criteria. The online platform Qualtrics (Provo, UT, USA) was used to administer the questionnaire. Data was exported to IBM SPSS Statistics (Version 29) for analysis. Descriptive statistics were used to summarise response frequency and Related-Samples Friedman’s Two-Way Analysis of Variance by Ranks were used to compare ranked preferences. All statistical tests were two-tailed and considered significant at *p* < 0.050. Surveys where less than 75% questionnaire items were completed were excluded from the analysis.

### Qualitative data collection and analysis

Semi-structured telephone interviews were conducted to probe patient preferences for receiving information about the role of exercise in cancer care, specifically exploring what might influence their decision to consider exercise as part of their cancer care plan (Online Resource 3). A study-specific semi-structured interview guide was developed using the same process as the questionnaire. The process involved literature review, stakeholder consultation (including patients and clinicians), iterative review by the research team and pilot testing (described in Online Resource 1). Brief, evidence-based exercise information was provided verbally to participants prior to discussing the primary questions to inform them of the evidence underpinning exercise guidelines (Online Resource 3). This information was provided to standardise minimum knowledge of the role of exercise in cancer care so participants responded from the same base of information when considering the type, format, and timing of exercise information that would best suit their individual needs. Interview questions probed preferences for what exercise information matters to them and explored AACTT domains [44] as described above.

Interviews were conducted by an experienced qualitative interviewer (AB) not involved in the care of participants, audio recorded and transcribed verbatim by a third-party service. Transcripts were checked for accuracy (100%) and uploaded to NVivo (QRS International). Interviews were analysed using an interpretive description approach [46, 47]. Interpretive description is an approach to qualitative data analysis with a focus on facilitating understanding of experiences in health care. Two researchers (AB, AN) independently coded all deidentified transcripts using both inductive and deductive approaches to combine the key themes that emerged with the AACTT framework [44]. Data were read and organised into meaning units, dominant views were identified (inductive approach) and mapped to AACTT framework domains (deductive approach). Interpretation was discussed at each stage of the analytical process with discrepancies resolved by consensus among the multidisciplinary research team. Ongoing discussion and refinement of data was conducted throughout the analytical process to ensure all perspectives were captured and the findings reflected both theory-driven and data-driven insights.

### Quantitative and qualitative data integration

Quantitative and qualitative data were integrated through a merging approach [48]. Datasets were brought together after analysis for comparison with areas of convergence and divergence explored [48].

## Results

### Demographics

Five hundred and seventy-three people initiated a questionnaire of which 109 were excluded due to completing ≤ 75% of the questionnaire items (30 ± 16% items completed). Eight were excluded due to being greater than 3 years after completing their most recent course of cancer treatment. A total of 456 people with 42 different types of cancer completed questionnaires (Table 1; 97 ± 5% items completed), from which a random sub-set of 30 participants were interviewed (Online Resource 4). Participants were 58 ± 13 years, 62.7% female, 2.1 ± 2.3 years since diagnosis, 64.9% currently receiving disease modifying treatment, 79.8% located in metropolitan areas, 64.7% tertiary educated, 39.3% currently employed, 46.3% ≥ $67,600 AUD annual income, 8.3% spoke a language other than English at home and 24.8% self-reported adhering with physical activity guidelines (Table 1). The relatively higher socioeconomic status and low representation of culturally and linguistically diverse patients may not be representative of all patients with cancer. Results should be interpreted accordingly as preferences of patients with the poorest cancer experiences may not be represented.

**Table 1:** Participant characteristics

People with cancer (*n* = 456)	*n*	%
	Mean ± SD	(range)
Age (years)	58 ± 13	(26–98 years)
Gender		
Female	286	62.7
Male	167	36.6
Cancer		
Breast	149	32.7
Blood	77	16.9
Prostate	56	12.3
Gynaecological	43	9.4
Bowel	25	5.5
Melanoma	24	5.3
Other	82	18.0
Time Since Diagnosis (years)	2.1 ± 2.3	0–17.7
Currently Receiving Treatment	296	64.9
Time Since Finishing Treatment (years)	1.0 ± 0.8	0–3
Treatments (previous and current)		
Surgery	313	68.6
Chemotherapy	295	64.7
Radiation	216	47.4
Hormone Therapy	140	30.7
Immunotherapy	116	25.4
Other	86	18.9
General Health Level (0—worst to 100—best health you can imagine)	71.9 ± 18.1	(2–100)
Language Other Than English Spoken at Home	38	8.3
Current Smoker	14	3.1
Married/Living with a Partner	334	73.2
Children Living at Home	135	29.6
Employed (full-time or part-time)	179	39.3
Education		
High School	60	13.2
University degree	167	36.6
Postgraduate university degree	128	28.1
Geographical Location		
Metropolitan	364	79.8
Regional	87	19.1
Income (combined pre-tax household annual income)^a^		
< $20,799 (< $399/week)	41	9
$20,800–$41,599 ($400–$799/week)	57	12.5
$41,600–$67,599 ($800–$1299/week)	57	12.5
$67,600–$103,999 ($1300–$1999/week)	81	17.8
$104,000 or more ($2000 or more/week)	130	28.5
Prefer not to say	83	18.2
Meet Exercise Recommendations (self-report)		
Yes (≥ 150 min aerobic & ≥ 2 resistance exercise sessions per week)	113	24.8
No	341	74.8
Previously Participated in a Cancer-Specific Exercise Program	155	34.1

^a^ Income in Australian dollars (AUD)

Below a synthesis of data from the questionnaire and interview responses are reported against the AACTT domains.

### What information would help patients to consider exercise – action domain

There were no standout benefits of exercise that participants considered most helpful in convincing them to exercise as part of their cancer care (Table 2). Over 75% of participants strongly agreed-to-agreed that information about 18 different types of potential benefits of exercise would help convince them to exercise during/after cancer treatment. Three percent of participants (*n* = 14) strongly agreed-to-agreed that no potential benefit of exercise would convince them to exercise during/after cancer treatment.

**Table 2:** What information messages would influence cancer patients’ thoughts about exercising as part of their cancer care plan

	Strongly Agree (%)	Agree (%)	Somewhat Agree (%)	Neither agree or disagree (%)	Somewhat Disagree (%)	Disagree (%)	Strongly Disagree (%)
Improve fitness and strength	59.3	31.1	7.0	1.9	0.2	0.2	0.2
Improve ability to cope with treatments^a^	58.7	26.7	8.8	3.8	1.0	0.7	0.2
Improve energy and stamina	58.4	33.2	6.1	1.4	0.2	0.5	0.2
Potential reduced risk of recurrence or mortality^b^	56.8	24.5	5.9	9.0	1.2	1.7	1.0
Prevent or reduce fatigue	54.9	32.3	9.5	1.6	0.9	0.5	0.2
Improve ability to do things that matter to me	54.6	35.8	6.6	1.9	0.5	0.5	0.2
Improve ability to think clearly and focus	54.2	31.9	8.5	4.2	0.5	0.2	0.5
Prevent or reduce declines in physical function	53.6	34.2	7.5	2.6	0.9	0.2	0.9
Feel more like myself and less like a cancer patient	53.4	32.2	8.0	3.3	1.9	0.7	0.5
Improve mood and feelings of happiness	52.2	37.0	6.5	2.3	0.5	0.9	0.7
Maintain/gain a sense of normality	51.6	33.5	8.6	3.7	0.9	0.7	0.9
Reduce feelings of depression and/or anxiety	50.6	35.1	8.5	4.0	0.5	1.2	0.2
Prevent or reduce severity of treatment side-effects	50.2	33.5	9.4	3.3	0.9	1.9	0.7
Potential reduced risk of another cancer diagnosis^c^	49.9	25.8	8.6	11.7	1.7	1.4	1.0
Feel a sense of control over the potential side effects	47.5	33.2	11.1	5.9	0.9	0.9	0.5
Improve confidence and self-esteem	44.8	34.5	11.3	5.9	1.6	1.2	0.7
Help manage other health conditions	44.7	35.6	9.6	7.0	0.5	1.9	0.7
Prevent or reduce distress	41.8	35.5	13.6	5.8	1.4	1.4	0.5
Already exercising so don’t need to be convinced	30.0	19.4	13.8	15.0	8.9	9.1	3.7
Nothing would convince me to exercise	2.2	1.2	1.7	2.6	5.3	19.4	67.6

^a^ i.e. can tolerate treatment side-effects better, may help prevent the need for treatment breaks;

^b^ Relevant for people diagnosed with breast, bowel/colon and prostate cancer;

^c^ Relevant for those diagnosed with breast, colon, endometrial, kidney, bladder, esophageal, or stomach cancer

A view commonly expressed in the qualitative interviews was that if exercise information was targeted and personalised it would be most helpful in influencing participants to exercise during/after cancer treatment (Table 3). A range of benefits were identified as motivating factors to exercise with the common theme being information that was person-centred and linked to what mattered to the participant whether that be counteracting symptoms or helping achieve their goals. Another view commonly identified by participants was that they were more likely to be convinced to exercise when it was described as important to their cancer care. Participants identified generic, non-cancer specific exercise information (i.e. exercise is good for everyone) as ineffectual. However, if exercise was framed as important in their cancer care or presented to them using evidence, participants were likely to feel more convinced to exercise during/after treatment.

**Table 3:** Data from qualitative interviews (*n* = 30) mapped to AACTT framework domains [42] for recommending exercise to cancer patients

AACTT Domain	Inductive Themes	Illustrative Quotes
Action (receiving information on exercise)	Targeted, personalised exercise information	“The fact that it was going to be personally tailored to me … but also you know cancer recovery really, really it really spoke to me as being something that would be likely to be incredibly effective.” – 60-year-old female with breast cancer
		“It’s got to be very individualised. It can’t be holistic for everybody, there’s too many variables.” – 41-year-old female with cervical cancer
	Cancer specific exercise information	“I think that they [doctor/nurse] need to show that it’s important, not be flippant about it or you know oh yeah maybe you should do a bit of exercise or something like that. They should be more oh yeah you definitely really should be doing it, it’s very beneficial in your situation, like come across you know quite seriously about it. If I had that happening to me I’d be listening” – 58-year-old male with leukaemia
		“For me it was like this is something that will improve the efficacy of the treatment, it will improve how you go through the treatment, it may mean that we are able to give you more chemo and for longer, because you’re fit able to handle that, and that can change your outcome. So for me what I would’ve appreciated was rather than just oh you should exercise, it’s a good idea to exercise, to talk through the outcomes, like you won’t be as fatigued, all of that stuff.” – 41-year-old female with breast cancer
Action (receiving information on how to access specialist care)	Referral to a qualified exercise specialist to provide an exercise program	“A set out program. Yeah and tailored individually for you. You know it would have to deal with your age and your capability and you know all that sort of stuff. I wouldn’t go with any sort of personal trainer, it would have to be someone very specialised in training people with cancer” – 47-year-old female with small bowel adenocarcinoma
		“I just think having the information there, of what you can do … I think it’s once you have someone there that can explain the exercise and this is what we’re going to do, that’s what really got me motivated.” – 65-year-old male with prostate cancer
Actor (who should provide exercise information)	Cancer care team	“I would’ve liked a conversation with the oncologist to say ‘alright you’re undergoing treatment and this is how it will impact you … and you should exercise’. Because people take their advice as gospel so I think the advice from an oncologist is really important” – 54-year-old female with melanoma
		“I think if the whole medical team were on board with that messaging around you know getting up and exercising and doing things I think that would be valuable and so I don’t think it needs to be necessarily just one person but more so a whole team” – 45-year-old male with acute myeloid leukemia
	If exercise wasn’t mentioned by the cancer care team, it was interpreted as unimportant	“No, nobody did [mention exercise], which is a shame … I think on the days that I felt alright I would’ve made an effort to do it but I just thought I was trying to take care of myself by not pushing myself too hard” – 44-year-old female with lymphoma
		“It’s really surprising actually that you do read so much about how exercise helps with cancer… and then when you actually go to see the doctor and nothing gets spoken about” – 61-year-old female with cervical cancer
Context (what format should exercise information be provided)	Conversation with cancer care team	“I prefer to hear from people especially the doctors. I mean you hang on every word that they say, you know like it’s really important what they tell you. Where there’s so much written information yeah and also with websites you know I forget that easier. Like I remember more what the doctor tells me than just reading stuff” – 61-year-old female with cervical cancer
		“I think probably from my haematologist, he’s the number one guy that looks after me, he’s the person I take the most notice of. … Pamphlets would be, look as I say a small percentage of people that really wanted to exercise and were already into that, would look. The rest of us couch potatoes I don’t think would look” – 58-year-old male with chronic lymphocytic leukemia
	Trusted information resources provided to supplement a conversation (i.e. not in lieu of a conversation)	“I think if you’re getting research from well-known sources rather than just searching yourself, it certainly is much more credible…you read so much rubbish on the internet, you’re not sure what’s fact and what’s fiction. So you know I just basically used what’s given to me” – 68-year-old male with myeloma
		“I’d been given a booklet by I think it was a nurse, like it was in a pack, it was about cancer and exercise or something, and I’m not even sure if I read it, because there just was so much information, I was quite overwhelmed.” – 44-year-old female with lymphoma
Time (when should exercise information be provided)	No consensus on the optimal time for exercise to be recommended	“Probably at diagnosis, well probably in the earliest conversations because … I’d be doing anything at that stage to help myself. Because once the treatment started, like once I had surgery and everything started to go badly, I had you know, I was already out of the habit of exercising” – 44-year-old female with lymphoma
		“Definitely not early on because early on is far too stressful, there was far too much going through my head, just a massive turmoil whirlwind emotional everything … You’d need to let it settle down before you really start talking about it. Because people need time in a cancer diagnosis, I would think, to allow themselves to settle enough to absorb the information” – 58-year-old male with leukaemia
	Continually discuss exercise throughout the cancer timeline	“Throughout the whole journey of when you get diagnosed and then the treatment begins and then you know you’re recovering from treatment. I think along that whole spectrum it would be great for someone just to mention the importance of exercise and the value that exercise brings.” – 45-year-old male with acute myeloid leukemia
		“If exercise was brought up consistently sort of mentioned a couple of times throughout appointments, like different appointments, that would’ve been convincing or useful or sort of cemented the fact that oh okay this is something that’s important” – 53-year-old male with melanoma

AACTT – Action, actor, context, target, time (AACTT) framework for behaviour specification of healthcare providers

Target domain – cancer patients

The information actions participants perceived to be most helpful from their cancer care team were referrals to a cancer-trained exercise specialist and cancer-specific exercise program (Table 4; 95.4% [*n* = 411] of participants considered a referral to be helpful). Having a trained exercise specialist to provide the exercise prescription and cancer-specific exercise information was commonly identified during interviews as the most helpful resources participants could receive from their cancer care team (Table 3). Participants described feeling unsure of what exercise after a cancer diagnosis involved and expressed a desire for detailed, individualised, cancer-specific guidance on what to do and how to do it. Being able to seek out this information from a health professional who specialises in cancer-specific exercise was consistently perceived as helpful. Participants also wanted help in accessing detailed information and a tailored program of exercises specific to their cancer experience from knowledgeable health professionals.

**Table 4:** What exercise information provision actions/resources would be considered helpful by cancer patients

	1 Very Much (%)	2 (%)	3 A Fair Bit (%)	4 (%)	5 Somewhat (%)	6 (%)	7 Not at All (%)
Referral to a cancer trained exercise specialist	76.6	10.9	7.9	1.9	1.4	0.7	0.7
Referral to a cancer specific exercise program	71.8	16.0	7.6	2.5	0.2	1.2	0.7
Written exercise recommendations from my cancer doctor or nurse	54.3	19.3	17.4	3.9	3.2	0.9	0.9
List of exercise options	51.5	23.2	17.5	3.8	2.4	1.4	0.2
List of the benefits of exercise for people with cancer	48.3	22.2	18.2	5.1	4.2	1.4	0.7
Information on the cost of exercise services	38.3	21.8	16.4	8.5	8.2	2.1	4.7
Video of exercises that are beneficial	37.9	23.1	21.2	6.6	7.5	1.9	1.9
Information website	34.0	27.5	24.1	5.3	6.3	1.7	1.2
Stories of other people with cancer who have started exercising	31.9	22.5	21.8	6.5	9.5	4.2	3.7
Information brochure	24.8	25.5	25.5	9.4	9.6	3.7	1.4
Brief information postcard	14.0	18.3	24.5	13.6	19.3	5.2	5.0

In summary, patients with cancer preferred to receive information on exercise that was personalised, cancer specific and supported by referral to a qualified exercise specialist.

### From whom patients would like to receive exercise information – actor domain

The most preferred person patients with cancer wanted to receive exercise information from was their cancer doctor (i.e. surgeon, medical oncologist, radiation oncologist, haematologist; Table 5). Patients ranked receiving information from their cancer doctor, exercise specialists and cancer nurses significantly higher than from a cancer patient who has been exercising (Table 5). The majority of participants surveyed would be influenced to consider exercise as part of their cancer care plan by their cancer care teams advice. Two thirds of participants reported that they would be ‘very much’ influenced by their doctor (67.1%, *n* = 306) or nurses’ (61.6%, *n* = 279) recommendation with 1.1–1.3% (*n* = 3–6) stating they would be ‘not at all’ influenced (Online Resource 5).

**Table 5:** Cancer patients’ preferences for the format they would like to receive information about the role of exercise in cancer care

	Mean Rank (mean ± SD)	Rank 1 (most preferred; %)	Rank 2 (%)	Rank 3 (%)	Rank 4 (%)	Rank 5 (%)	Rank 6 (%)	Rank 7 (%)	Rank 8 (least preferred; %)
Discussion with cancer doctor	2.7 ± 1.8	30.8	28.1	15.8	11.2	4.6	4.0	2.9	2.6
Discussion with exercise specialist	2.8 ± 1.6	26.6	22.6	23.1	11.9	7.9	5.1	2.0	0.9
Discussion with cancer nurse	3.6 ± 1.7*	4.2	22.4	33.4	16.9	7.5	7.3	5.7	2.6
A combination of all options	4.1 ± 2.5*°	27.9	8.8	4.2	11.4	16.5	10.1	8.8	12.3
Discussion with a patient who has been exercising	5.3 ± 2.0*°	2.4	6.8	8.6	19.8	18.5	10.8	15.2	18.0
Website	5.3 ± 1.8*°	3.7	4.6	7.3	12.3	21.3	22.4	19.6	8.8
Brochure	6.1 ± 1.9*°	3.1	3.5	4.2	10.3	10.5	18.0	20.9	29.5
Video	6.2 ± 1.7*°	1.3	3.1	3.5	6.2	13.2	22.4	25.1	25.3

^*^Significantly (*p* < 0.001) different from ‘discussion with cancer doctor’ and ‘discussion with exercise specialist’;

°Significantly (*p* < 0.001 to 0.003) different from ‘discussion with cancer nurse’;

Related-Samples Friedman’s Two-Way Analysis of Variance by Ranks

Interview participants described the importance of receiving exercise information directly from the cancer care team (Table 3). Participants specifically identified a strong desire to learn about the evidence-based exercise guidelines from their cancer care team, especially their doctors. Rationale provided for this viewpoint centred on the importance placed on advice received from doctors. Another common view was that if exercise wasn’t mentioned by the cancer care team, it was interpreted as unimportant or as permission to be inactive (Table 3). Many participants had not received any exercise information from their cancer care team. It was acknowledged that this was likely due to exercise being a low priority among the many things health professionals need to discuss with patients. Despite this acknowledgement, participants described feeling that exercise was unimportant to their cancer care when it was not raised by a member of their treating team. After learning about exercise oncology evidence-based guidelines, participants said that they would have liked to have received exercise advice and that they would have likely taken it under advisement (Table 3).

In summary, patients with cancer preferred to receive exercise information from their cancer care team and would be likely to act on their advice, especially when provided by their doctor.

### How patients would like to receive exercise information – context domain

The most preferred format to receive information was through discussions with health professionals (Table 5). Almost 62% of participants (*n* = 280) ranked discussions with a cancer doctor, exercise specialist or cancer nurse as the most preferred format of information provision. Discussions with these health professionals were significantly (*p* < 0.001) more preferred than other options for provision of exercise information including online/print materials (Table 5). Furthermore, approximately half of participants considered written exercise recommendations received from their cancer doctor or nurse (54.3%, *n* = 234) as well as a list of exercise options (51.5%, *n* = 218) and a list of exercise benefits (48.3%, *n* = 209) as very helpful (Table 4). Approximately a third of participants considered digital resources such as videos (37.9%, *n* = 161) and websites (34.0%, *n* = 141) as very helpful, while less than a quarter of participants considered print materials (e.g. brochures [24.8%, *n* = 106], postcards [14.0%, *n* = 59]) very helpful (Table 4).

Interviewed participants articulated a clear preference for exercise information to be conveyed through a conversation with their cancer care team, especially their doctors (Table 3). Participants reiterated their viewpoint that information from their cancer doctors (and nurses) holds much more weight and therefore is recalled more than online/print information. It was also identified that information conveyed through a conversation would be more specific to their individual circumstances (i.e. cancer type, treatments, symptoms, etc.) rather than generic information provided in print/online materials. However, participants indicated that print/online resources would be valued if they were provided by their cancer care team after a conversation has taken place. If provided in lieu of a conversation, participants were unlikely to read print/online resources but had a desire for supplementary information to support and extend a conversation with their cancer care team. Furthermore, these information sources would be trusted rather than the patient having to attempt to locate credible cancer-specific exercise information themselves.

In summary, patients preferred to receive exercise information through a conversation with their cancer care team, supplemented by trusted information resources.

### When patients would like to receive exercise information – time domain

The preferred time to receive information about exercise was early in the treatment continuum (Table 6). Seventy percent of participants (*n* = 320) most preferred to learn about the role of exercise earlier in their cancer care, 23.6% (*n* = 107) regularly and 5.7% (*n* = 26) during/after treatment. Receiving exercise information shortly after being diagnosed and before starting treatment were significantly more preferred than all other timepoints (*p* < 0.001).

**Table 6:** When cancer patients would like to receive information about the role of exercise in cancer care

	Mean Rank (mean ± SD)	Rank 1 (most preferred; %)	Rank 2 (%)	Rank 3 (%)	Rank 4 (%)	Rank 5 (%)	Rank 6 (%)	Rank 7 (least preferred; %)
Shortly after being diagnosed	2.7 ± 2.1	41.1	20.8	9.5	6.8	6.6	4.6	10.6
Before starting treatment	2.6 ± 1.4	19.9	34.2	29.4	6.6	4.4	3.1	2.4
At the start of treatment	3.3 ± 1.3*	9.7	11.7	34.0	32.7	7.1	3.1	1.8
Regularly	3.4 ± 2.1*	23.6	23.8	8.6	15.0	9.7	3.5	15.7
During treatment	4.3 ± 1.2*°	3.1	4.4	12.8	29.8	43.7	3.8	2.4
At the end of treatment	5.4 ± 1.1*°	1.3	3.5	2.9	5.5	23.0	59.4	4.4
After the end of treatment	6.3 ± 1.3*°	1.3	1.5	2.9	3.5	5.5	22.5	62.7

^*^Significantly (*p* < 0.001) different from ‘shortly after being diagnosed’ and ‘before starting treatment’;

°Significantly (*p* < 0.001) different from ‘regularly’ & ‘at the start of treatment’;

Related-Samples Friedman’s Two-Way Analysis of Variance by Ranks

When interviewed, participants had different views about optimal time to receive exercise information (Table 3). Learning about exercise close to diagnosis was strongly preferred by some participants due to their eagerness to do everything they could to improve their cancer experience. Additionally, they expressed a feeling of capability to start an exercise routine that could be continued throughout treatment rather than trying to initiate exercise during/after treatment while dealing with the additional barriers of treatment-related side effects. Contrasting perspectives were observed where learning about exercise at the time of diagnosis was not preferred due to feeling too overwhelmed by the cancer diagnosis to absorb information about exercise. Many participants stated that exercise should be continually discussed across the cancer pathway. Participants identified that hearing information about exercise multiple times throughout their cancer experience would cement it as something important in their care.

In summary, most patients in this study reported a preference for information about exercise to be introduced early in the care continuum and reiterated regularly. However, being informed about exercise too close to their diagnosis was least preferred by some patients.

## Discussion

**Fig. 1:**
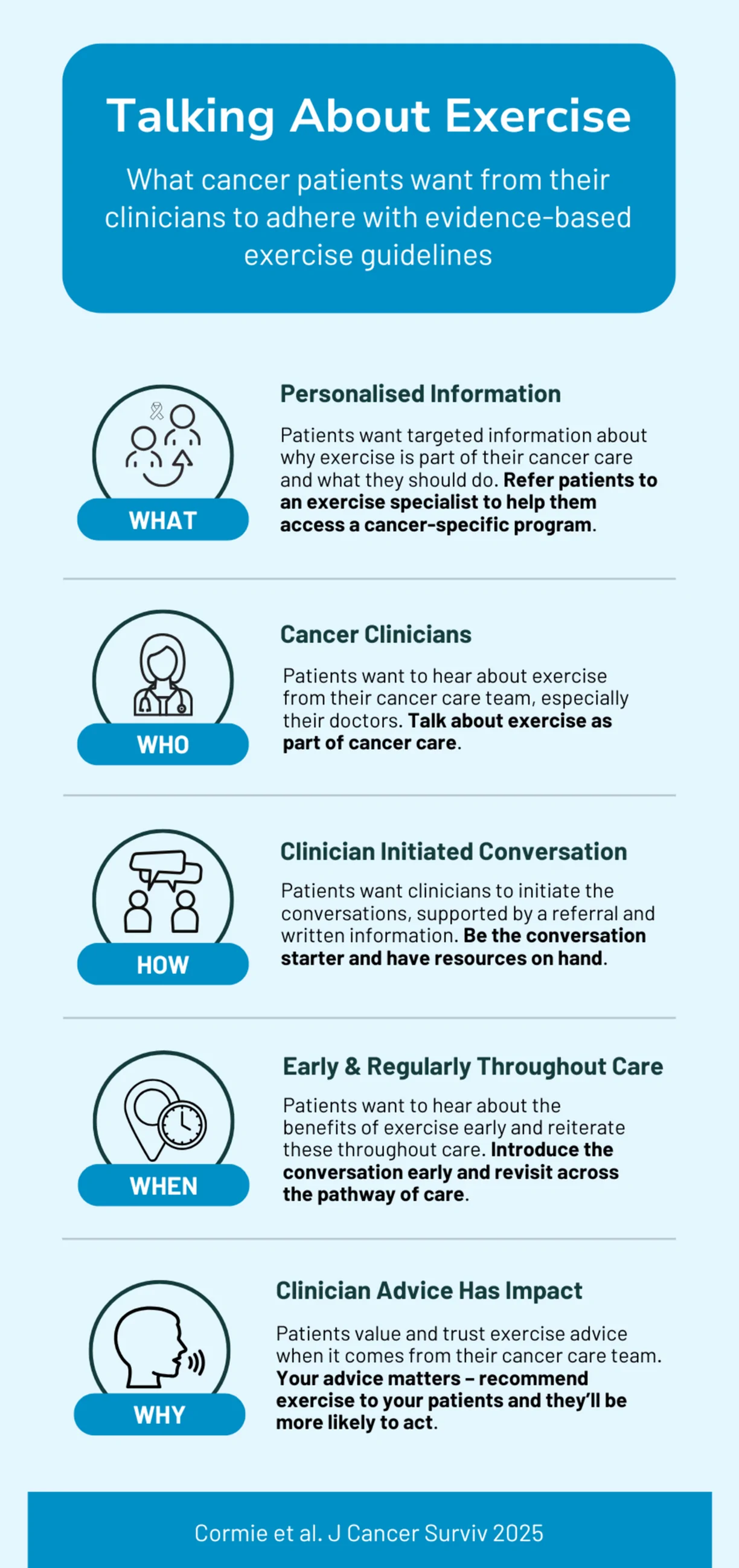
Quick reference guide for healthcare professionals

Evidence-based guidelines advise cancer clinicians to recommend exercise to patients in order to mitigate cancer and treatment-related side-effects [6–12]. While previous work provides insight into screening and referral aspects of exercise recommendations [22–28], cancer clinicians have little pragmatic guidance on how to initiate conversations about exercise and how exercise recommendations can be aligned with patient needs and preferences. This study begins to address this knowledge gap by identifying that patients would be more likely to consider exercise as part of their cancer care plan if information was provided during a discussion with their cancer clinician, introduced early in the care continuum and reiterated often, using personalised and cancer-specific messaging that was supported by referral to cancer-specific exercise services (Fig. 1).

Participants in this study identified a clear preference for receiving information about exercise through discussion with their clinicians rather than via print/online content. This was underscored by the finding that if exercise went unmentioned by cancer clinicians, patients perceived exercise to be unimportant in their care or interpreted the omission as permission to be inactive. The potential of clinicians to modify exercise attitudes and beliefs of patients has been defined as a factor that can either facilitate or inhibit exercise intentions when information is provided or absent from consultations [32–36]. Data from this study quantifies the extent to which clinician advice impacts patient behaviour, with over 90% (*n* = 430) of cancer patients surveyed being influenced to exercise by their cancer doctors or nurses’ advice, and only 1% (*n* = 6) of patients not at all influenced. Participants attributed this level of influence to the authority and credibility of cancer clinicians which is in line with previous research linking trust in practitioners with treatment adherence [29, 30]. Clinician-initiated discussions about exercise were desired, supporting previous evidence that 86% of patients with cancer thought it was the “doctor’s duty” [49] to provide physical activity advice (data from a survey of 222 adults with a cancer diagnosis in the United Kingdom [49]). Despite the challenges to discussing exercise in time-constrained clinical consultations [19, 20], results from this study indicate that doing so would align with patient preferences, and likely influence exercise intentions.

Clinicians have been encouraged to capitalise on teachable moments during a cancer experience [50] but in the absence of evidence-informed strategies, are left to make judgements about when is the optimal time to recommend exercise [51]. Our data indicated that earlier in the cancer continuum is the most preferred time to receive information about exercise. There was also a preference for exercise information to be provided regularly as patients explained that hearing about exercise multiple times during their care would help establish exercise as an important component of their treatment. Collectively 94% (*n* = 427) of patients most preferred to receive exercise information early and regularly throughout the care continuum, but these options were also least preferred by some patients (i.e. 11% [*n* = 48] least preferred shortly after diagnosis). There was a contrast between the rationale of wanting information early when patients felt eager and willing to do whatever they could to get through cancer, and feeling too overwhelmed to retain information or act on advice. It is unclear if preferences for timing of receiving exercise information matches to the time in which patients may be most amenable to acting on the recommendation. Previous work has described patient preferences for exercise programs at various times throughout the cancer care continuum with a tendency towards after finishing treatment [32–35]. It is unclear how an early recommendation from a clinician would influence the theoretical constructs underpinning behaviour change and whether patients would exercise sooner than they may have otherwise preferred [36, 37]. Preferences for receiving exercise information early in the care continuum corresponds with physiological rationale for early exercise intervention to mitigate treatment-related side effects [1–12, 52–54].

Various evidence-based benefits of exercise can be used to inform patients about the role of exercise in cancer care [1–12, 52–54], but it is unclear what information matters most to patients. This study observed cancer patients may be persuaded to exercise by a vast array of messages, with only 3% (*n* = 11) indicating nothing would convince them to exercise. There was no clear preference for certain exercise benefits as motivators to exercise. Patients instead identified targeted, personalised information to be most compelling. Our data indicate that it appears unlikely that generic exercise advice will prompt adherence, rather, behaviour change may be more likely when exercise information is linked to what matters most to each patient [31, 55]. Patients also identified that exercise recommendations were ineffectual when delivered using generalised statements (e.g. be active/keep up your exercise). If exercise was framed as important in their cancer care participants felt more convinced to act on the information. Thus, when deciding what to say when recommending exercise, providing individualised cancer-specific information is most likely to align with patient preferences. The ability of clinicians to navigate a personalised conversation about exercise is expected to impact patient adherence, as meta-analyses indicate the odds of patient adherence are 2.16 (95% confidence interval 1.91–2.35, *p* = 0.001) times higher if their clinician is a skilled communicator [29].

A key question to capitalising on clinician exercise recommendations is what actions do patients want/need from their cancer care team to help them adhere with exercise guidelines. Receiving referrals to a cancer-specific exercise specialist or program were most preferred by patients. Patients described a desire for expert guidance from a qualified health professional that is personalised and cancer-specific rather than having to figure it out by themselves. These preferences align with the strong emphasis throughout the literature for the development of exercise oncology programs and referral guides [1–12, 22–28, 52–54]. Patients also valued a written exercise recommendation from their doctor and/or nurse. While patients described wanting detailed information on what exercises to do for their individual circumstances, they acknowledged that to be outside their cancer doctor/nurse scope of practice. It’s possible that an overarching written recommendation proactively provided by their clinician was desired to aid patient recall of verbal advice and substantiate the clinician’s endorsement of exercise being important in their cancer care [39, 56]. This content should not be likened to information brochures/postcards which were least preferred by patients. Patients discussed being inundated by brochures to read and disliking generic information, but still acknowledged the value of the information they contain.

Several limitations to this study should be considered when interpreting the findings. The self-selecting sample of participants introduce bias through an implied interest in exercise and/or research. Participants were proficient in English and had relatively higher socio-economic status that may not be reflective of the broader cancer population. Thus, reported outcomes among culturally and linguistically diverse patients and groups with the poorest cancer experiences are unclear. Future research is required to better understand patient preferences for exercise information at different timepoints across their disease and treatment trajectory, and importantly to explore the preferences of people from diverse backgrounds for information and support to engage with positive health behaviours such as exercise.

When faced with the challenge of implementing exercise recommendations in routine cancer care, clinicians have been disadvantaged by a lack of evidence regarding how and when information should be given, what information matters to patients and what actions would help patients adhere to evidence-based guidelines. This study provides new data to address these questions and help clinicians deliver exercise recommendations that are informed by and responsive to patient needs and preferences (Fig. 1). While only one element of the multifaceted implementation effort required to translate exercise oncology evidence into practice [57, 58], this new knowledge allows for the development of pragmatic tools and strategies to support the skilled provision of exercise information and optimise the effectiveness of exercise recommendations. The next step in this body of work is to determine the unmet needs and preferences of cancer clinicians to encourage uptake of evidence informed exercise among patients in their care. Our team is currently preparing a publication reporting on these important perspectives.

## Supplementary Information

Below is the link to the electronic supplementary material.

ESM 1 (DOCX 29.0 KB)

## Data Availability

The datasets generated during the current study are available from the corresponding author on reasonable request.
