# Supplementary material for: What patients with cancer require from their clinicians to enable uptake of exercise as part of cancer care: A mixed methods study
Source: J Cancer Surviv. 2025 Mar 25;20(Suppl 1):1–15. doi: 10.1007/s11764-025-01779-4 (PMC13676566; doi:10.1007/s11764-025-01779-4)
Supplement: Supplementary file 1 [file 11764_2025_1779_MOESM1_ESM.docx]

**SUPPLEMENTAL TABLES**

**Online Resource 1**. Process involved with questionnaire and semi-structured interview development

1. Lead investigator reviewed the literature to identify current knowledge gaps.
2. Lead investigator conducted interviews with 5 cancer patients and 20 cancer clinicians to inform clinical and consumer perspectives of available literature and knowledge gaps.
3. Lead investigator drafted questionnaire items and semi-structured interview guide.
4. Questionnaire items and semi-structured interview guide were reviewed and refined by research team which included cancer clinicians and cancer patient consumer representatives.
5. Questionnaire items and semi-structured interview guide were reviewed by 8 cancer nurses working across different cancer types in a specialist cancer hospital in order to obtain a broad perspective of patient needs expressed in routine practice. Feedback was collected through think aloud methodology [45] which informed refinements.
6. Questionnaire items underwent pilot testing with 8 patients who had various types of cancer with feedback gathered from interviews using think aloud methodology [45] guiding enhancements.
7. Final versions of the questionnaire items and semi-structured interview guide were reviewed and approved by the research team.

**Online Resource 2**. Questionnaire pre-amble and custom items

**Background information on exercise provided to participants prior to completing the questionnaires**

What benefit does exercise have for people with cancer?
Based on what research tells us, people with cancer who exercise have:
 • fewer and less serious side effects (e.g. less tiredness, less distress, better fitness);
 • better physical and mental health;
 • and better quality of life than people who do not exercise during or after their cancer treatment.

People with cancer involved in exercise programs talk about:
 • how exercise helped them feel more like themselves and less like a patient;
 • helped them to keep doing the things that were important to them; and
 • gain a sense of control and normality.
This video shows people with cancer talking about some other benefits they had from an exercise program: https://youtu.be/2rCV0KnET4A

Exercise isn’t the same for everyone
Exercise can be changed and modified so that it is at the right level for each person no matter how sick or well they are. Exercise is customized to suit every person’s individual needs. Changes are made based on the type and stage of cancer, any side effects experienced and overall health. There are many ways to exercise including in the gym, at home or around your local area.

**Questionnaire items specifically developed for this study**

• What information would help convince you to exercise during/after your cancer treatment? I would be convinced by messages that explained that exercise can:

• How would you like to receive information about the role of exercise in your cancer treatment plan?
• How much would your [option] advice about exercise influence your thoughts about exercising as part of your cancer treatment plan?
 o [option] = 1) cancer doctor’s (specialist); 2) cancer nurse's; 3) hearing from cancer patients who have exercised during/after treatment
• When would you like to/have liked to receive information about exercise with your cancer doctors (specialists) and nurses?
• Below is a list of resources you could receive from your cancer doctor (specialist) or nurse about exercising with cancer. How helpful would you find these
 things?
• Is there anything we’ve missed that you’d like us to know? Anything else about the way you’d like to receive information about exercise from your cancer
 doctors and nurses?

**Online Resource 3**. Semi-structured interview guide pre-amble and primary questions

**Background information on exercise provided to participants prior to discussing the primary questions**

I’d like to share a couple of research-based facts with you that will help you respond to the questions I’ll ask you today. They are for context only, and there are

no right or wrong answers. Feelings about exercise are very individual so please feel able to speak as freely as you want.

People with cancer who exercise report fewer and less severe treatment-related side effects (e.g. less fatigue, less physical decline, experience less distress),

better physical and mental wellbeing, and better quality of life than people who do not exercise during or after their cancer diagnosis. People with cancer who have been involved in exercise programs talk about how exercise helped them feel more like themselves and less like a patient; helped them to keep doing the things that were important to them, as well as gain a sense of control and normality.

**Primary questions developed for this study (semi-structured format)**

• Are there any potential benefits of exercise that matter or appeal most to you. Why do they matter most to you?

• Would anything prompt you to consider exercise as part of your cancer treatment plan? If so, what? Why would these things be helpful?

• Is there any particular member of your medical team whose advice about exercise would have the most influence on your decision to exercise or not? Why?

• Is there anything your doctor/nurse could say/could have said to you that would help you consider exercise as part of your cancer treatment plan? Why/why
 not would their advice influence your thinking about exercise?

• Is there any particular time before, during or after your treatment that you would/would have liked your doctor/nurse to discuss exercise with you? Why would
 you prefer that time?

• Do you think that having some resources or information would help/would have helped you consider exercise as part of your cancer treatment? If so, what?

Why/why not would these things be helpful?

**Online Resource 4**. Participant characteristics for sub-set of participants who completed interviews

| **PEOPLE WITH CANCER (n = 30)** | **n** | **%** |
| --- | --- | --- |
|  | **Mean ± SD** | **(range)** |
| **Age (years)** | 59 ± 13 | (38-98 years) |
| **Gender** |  |  |
| Female | 17 | 56.7 |
| Male | 13 | 43.3 |
| **Cancer** |  |  |
| Breast | 8 | 26.7 |
| Blood | 9 | 29.9 |
| Prostate | 2 | 6.7 |
| Gynaecological | 2 | 6.7 |
| Bowel | 3 | 10.0 |
| Melanoma | 2 | 6.7 |
| Other | 4 | 13.3 |
| **Time Since Diagnosis (years)** | 1.8 ± 1.9 | 0-10.3 |
| **Currently Receiving Treatment** | 16 | 53.3 |
| **Time Since Finishing Treatment (years)** | 1.3 ± 0.8 | 0-2.8 |
| **Treatments (previous & current)** |  |  |
| Surgery | 20 | 66.7 |
| Chemotherapy | 18 | 60.0 |
| Radiation | 15 | 50 |
| Hormone Therapy | 7 | 23.3 |
| Immunotherapy | 6 | 20 |
| Other | 7 | 23.3 |
| **General Health Level (0 - worst to 100 - best health you can imagine)** | 71.6 ± 16.0 | (30-93) |
| **Language Other Than English Spoken at Home** | 1 | 3.3 |
| **Current Smoker** | 2 | 6.7 |
| **Married/Living with a Partner** | 22 | 73.3 |
| **Children Living at Home** | 8 | 26.7 |
| **Employed (full-time or part-time)** | 10 | 33.3 |
| **Education** |  |  |
| High School | 4 | 13.3 |
| University degree | 11 | 36.7 |
| Postgraduate university degree | 8 | 26.7 |
| **Geographical Location** |  |  |
| Metropolitan | 21 | 70 |
| Regional | 9 | 30 |
| **Income (combined pre-tax household annual income)^a^** |  |  |
| < $20,799 (< $399/week) | 2 | 6.7 |
| $20,800 – $41,599 ($400 – $799/week) | 3 | 10 |
| $41,600 – $67,599 ($800 – $1,299/week) | 6 | 20 |
| $67,600 – $103,999 ($1,300 –$1,999/week) | 5 | 16.7 |
| $104,000 or more ($2,000 or more/week) | 10 | 33.3 |
| Prefer not to say | 4 | 13.3 |
| **Meet Exercise Recommendations (self-report)** |  |  |
| Yes (≥150mins aerobic & ≥2 resistance exercise sessions per week) | 9 | 30.0 |
| No | 21 | 70.0 |
| **Previously Participated in a Cancer-Specific Exercise Program** | 12 | 40 |
|  |  |  |

^a^ Income in Australian dollars (AUD)

**Online Resource 5**. How much would cancer patients’ thoughts about exercising as part of their cancer care plan be influenced by advice

|  | **1 - Very Much**  **(%)** | **2**  **(%)** | **3 - A Fair Bit**  **(%)** | **4**  **(%)** | **5 – Somewhat**  **(%)** | **6**  **(%)** | **7 - Not At All**  **(%)** |
| --- | --- | --- | --- | --- | --- | --- | --- |
| **Cancer doctor’s (specialist) advice** | 67.1 | 13.8 | 13.4 | 2 | 2 | 0.4 | 1.3 |
| **Cancer nurse's advice** | 61.6 | 15.7 | 15.9 | 2.4 | 2.4 | 0.9 | 1.1 |
| **Cancer patients who exercised during/after treatment** | 49.7 | 18.3 | 18.3 | 2.6 | 7.5 | 2.2 | 1.3 |
|  |  |  |  |  |  |  |  |
